# 3-Methyl-5-methyl­sulfanyl-1,3,4-thia­diazole-2(3*H*)-thione

**DOI:** 10.1107/S1600536812040147

**Published:** 2012-09-29

**Authors:** Sebastian A. Suarez, Saroj K. S. Hazari, Biplab Ganguly, Fabio Doctorovich, Tapashi G. Roy, Ricardo Baggio

**Affiliations:** a Departamento de Química Inorgánica, Analítica y Química, Física/INQUIMAE-CONICET, Facultad de Ciencias Exactas y Naturales, Universidad de Buenos Aires, Argentina; b University of Chittagong, Chittagong 4331, Bangladesh; c Gerencia de Investigación y Aplicaciones, Centro Atómico Constituyentes, Comisión Nacional de Energía Atómica, Buenos Aires, Argentina

## Abstract

The title compound, C_4_H_6_N_2_S_3_, has two very similar mol­ecules per asymmetric unit. The nine non-H atoms in each mol­ecule are coplanar, both having comparable r.m.s. deviations of 0.002 Å. The main inter­est in the rather simple structure resides in a survey of very weak (in some cases, borderline) non-bonding inter­actions of various kinds, *viz*. S⋯S, C—H⋯π, π–π [centroid–centroid distance = 3.8958 (13) Å] and C—S⋯π [3.7271 (11) Å], which act as the major driving force for the arrangement of mol­ecules in the structure. The role of long, though highly directional, S⋯S contacts (*d* > 3.60 Å), and their relevance to the stability of the structure is discussed.

## Related literature

For the synthesis and characterization of the title compound, see: Espinosa *et al.* (2010 ▶); Thorn (1960 ▶). For the reactivity of thia­diazole, see: Espinosa *et al.* (2010 ▶). For significance of weak S⋯S inter­actions and for the role of weak inter­actions in the absence of stronger ones, see: Allen (2002 ▶); Bats (1976 ▶); Bondi (1964 ▶); Desiraju & Steiner (1999 ▶); Mrozek *et al.* (2000 ▶); Iwaoka & Isozumi (2012 ▶).
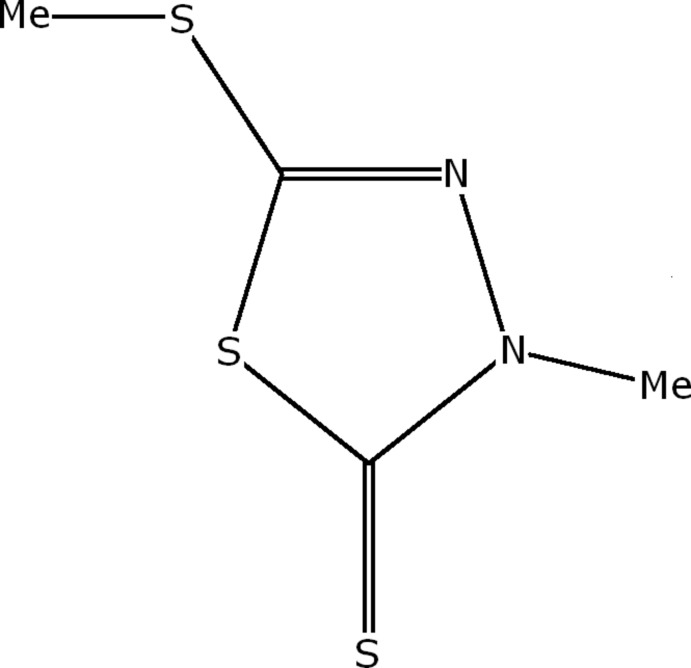


## Experimental

### 

#### Crystal data

C_4_H_6_N_2_S_3_
*M* *_r_* = 178.30
Monoclinic, 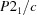

*a* = 9.3505 (4) Å
*b* = 22.4118 (9) Å
*c* = 7.6682 (5) Å
β = 106.661 (5)°
*V* = 1539.50 (14) Å^3^
*Z* = 8
Mo *K*α radiation
μ = 0.88 mm^−1^
*T* = 295 K
0.3 × 0.2 × 0.2 mm

#### Data collection

Oxford Diffraction Gemini CCD S Ultra diffractometer
Absorption correction: multi-scan (*CrysAlis PRO*; Oxford Diffraction, 2009 ▶) *T*_min_ = 0.81, *T*_max_ = 0.84
32965 measured reflections
3821 independent reflections
2690 reflections with *I* > 2σ(*I*)
*R*_int_ = 0.060

#### Refinement

*R*[*F* ^2^ > 2σ(*F* ^2^)] = 0.040
*wR*(*F* ^2^) = 0.102
*S* = 1.04
3821 reflections
167 parameters
H-atom parameters constrained
Δρ_max_ = 0.25 e Å^−3^
Δρ_min_ = −0.27 e Å^−3^

### 

Data collection: *CrysAlis PRO* (Oxford Diffraction, 2009 ▶); cell refinement: *CrysAlis PRO*; data reduction: *CrysAlis PRO*; program(s) used to solve structure: *SHELXS97* (Sheldrick, 2008 ▶); program(s) used to refine structure: *SHELXL97* (Sheldrick, 2008 ▶); molecular graphics: *SHELXTL* (Sheldrick, 2008 ▶); software used to prepare material for publication: *SHELXL97* and *PLATON* (Spek, 2009) ▶.

## Supplementary Material

Crystal structure: contains datablock(s) I, global. DOI: 10.1107/S1600536812040147/zl2506sup1.cif

Structure factors: contains datablock(s) I. DOI: 10.1107/S1600536812040147/zl2506Isup2.hkl

Supplementary material file. DOI: 10.1107/S1600536812040147/zl2506Isup3.cml

Additional supplementary materials:  crystallographic information; 3D view; checkCIF report

## Figures and Tables

**Table 1:** Selected interatomic distances (Å)

S3⋯S2^i^	3.6438 (10)
S2⋯S2^ii^	3.6319 (9)
S6⋯S5^iii^	3.7189 (11)
S3⋯S4^iv^	3.3671 (10)
S1⋯S6^v^	3.3621 (10)
S1⋯S5^iv^	3.8332 (11)
S2⋯S4^v^	3.8778 (10)

Symmetry codes: (i) 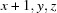; (ii) 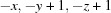; (iii) 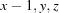; (iv) 
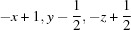
; (v) 
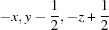
.

**Table 2:** C—H⋯π interaction (Å, °) *Cg*1 is the centroid of the C1,C2,N1,N2,S1 ring.

*D*—H⋯*A*	*D*—H	H⋯*A*	*D*⋯*A*	*D*—H⋯*A*
C4—H4*C*⋯*Cg*1^vi^	0.96	2.86	3.589 (3)	134

Symmetry code: (vi) 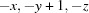.

**Fig. 1.**
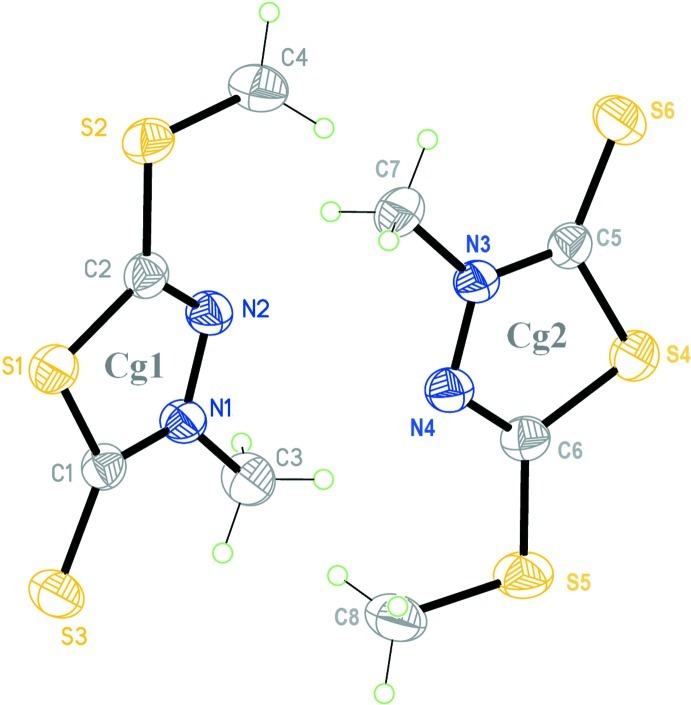
The molecular structure of the title compound showing the atom-labeling scheme and displacement ellipsoids at the 40% probability level.

**Fig. 2.**
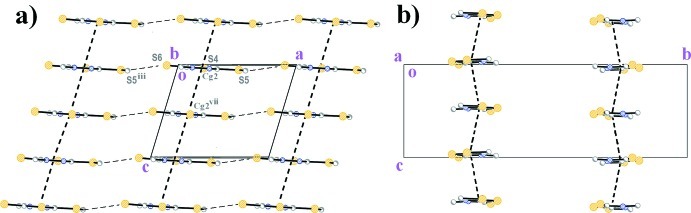
Crystal packing for the B substructure. a) viewed along b. b) viewed along a. Dashed lines indicate π–π bonds (See Table 3 for details). Symmetry codes: (iii) x-1, y, z; (vii) x, 3/2-y, 1/2+z.

**Fig. 3.**
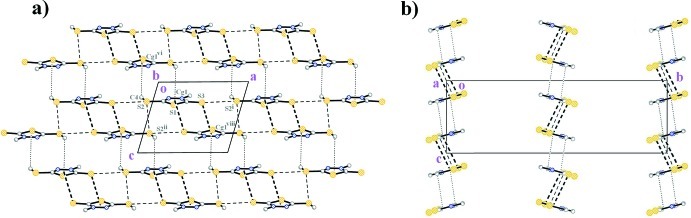
Crystal packing for the A substructure. a) viewed along b. b) viewed along a. Heavy dashed lines indicate S···π interactions; soft dashed lines indicate S···S interactions; dotted lines indicate C—H···π interactions; (See Tables 1, 2 and 3 for details). Symmetry codes: (i) x+1, y, z; (ii) -x, -y+1, -z+1; (vi) -x, -y+1, -z; (viii) 1-x, 1-y, 1-z.

**Fig. 4.**
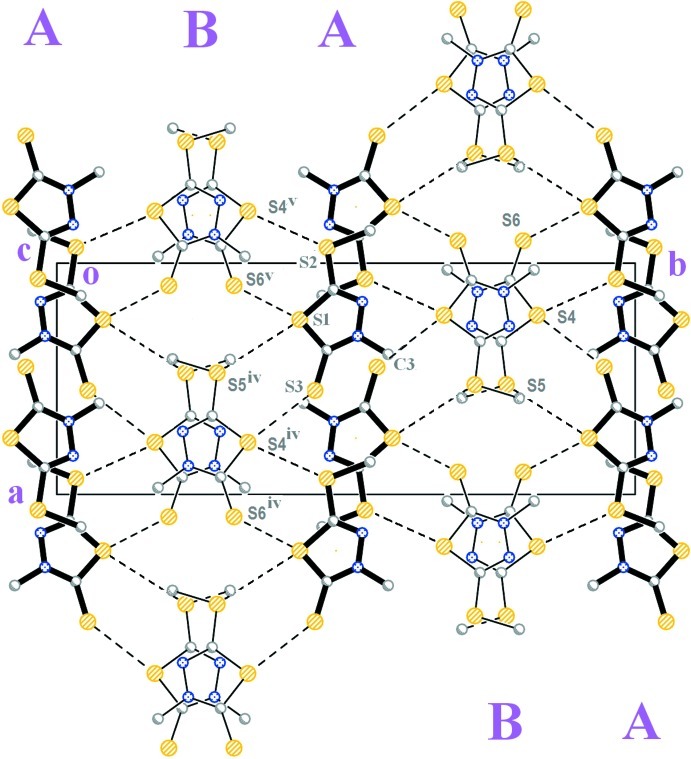
Complete crystal packing viewed along c and showing only interactions between A and B substructures. Soft dashed lines indicate S···S interactions (See Tables 1 for details). Symmetry code: (iv) -x+1, y-1/2, -z+1/2 (v) -x, y-1/2, -z+1/2.

**Fig. 5.**
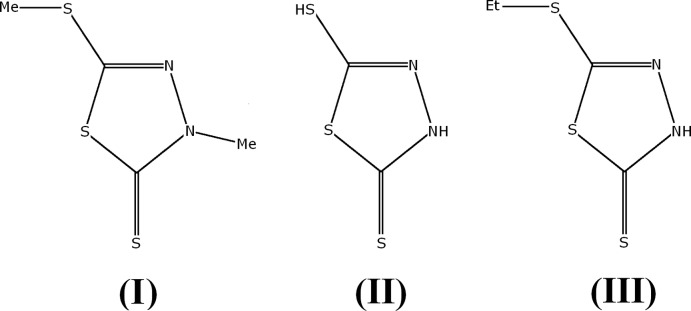
Molecular scheme of the three closely related structures compared in the paper.

## supplementary crystallographic information

### Comment

During a systematic trial intended to synthesize a molybdenum(VI) complex of our interest (see experimental section for details) excellent crystals were obtained, at the time thought to correspond to the expected product. A straightforward crystal structure determination disclosed that the compound was in fact the rather simple heterocyclic title compound, C_4_H_6_N_2_S_3_ (Scheme 1), a readily available commercial product (Thorn, 1960; Espinosa *et al.*, 2010), but the crystal structure of which had not been reported so far. Incidentally, the molecule has very little to do with any of the starting materials used, and the mechanism through which it could have been generated during the unsuccessful synthesis remains basically unclear (the point is further discussed in the experimental section). In addition to the rather expectable molecular information the study revealed a surprising collection of varied non-bonding interactions which affect the overall stability of the crystal structure, and to the analysis of which most of the following discussion will be devoted.

The asymmetric unit of the title compound includes two independent molecules (*A* and *B*) consisting of a 1,3,4-thiadiazole ring, with a methyl group attached at position 3 and a thiomethyl at position 5 (Figure 1). Both are essentially planar (max. deviations from planarity, 0.002 Å) but not parallel (dihedral angle: 14.87 (4)°).

The two independent molecules exhibit no significant differences between each other (RMS deviation from the L.S. fit: 0.0159 (3) Å, max. deviation: 0.026 (2) Å for the N1, N4 pair) nor show they deviations from commonly accepted values for either bond distances or angles (CSD, version 1.14, Allen, 2002). S—C bonds of different types are fairly distinct and present a remarkable homogeneity in both moieties, as assessed by the tight ranges displayed: <S—C_arom_>: 1.734 (2) -1.738 (2); <S═C_arom_>: 1.655 (2) -1.662 (2); <S—C_methyl_>: 1.796 (3) -1.797 (3).

With the molecular details being basically unexceptional, the most interesting aspect of the structure resides in its packing: in this respect this is a good example of very weak forces (London's, dipole-induced dipole, *etc*.) expressed as a variety of usually borderline interactions of various types (S···S (Table 1); C—H···π (Table 2); π–π, C—S···π (Table 3)) which in the absence of stronger ones can become the basic synthons promoting molecular recognition and intermolecular interaction, and thus playing an essential constructive role in the crystal lattice (Desiraju & Steiner, 1999; Iwaoka & Isozumi, 2012 (and references therein)).

Contrasting with the similarities shown by the internal molecular geometries, the packing behavior for the two independent moieties is quite different, for what they will be analyzed separately, molecule *A* (C1—C2—C3—C4—N1—N2—S1—S2—S3) and molecule *B* (C5—C6—C7—C8—N3—N4—S4—S5—S6).

Molecule *B* is the most simple to describe. Fig 2a shows its disposition in the crystal structure: the most relevant *B···B* interaction is a π–π contact between neighboring aromatic rings (Table 3: Entry 1, hereafter summarized in the compact expression T3:E1). These contacts link molecules into columnar arrays running along [001]. The *a* unit cell translation generates parallel columns *a*.sin(β) apart (~ 9 Å), thus defining some kind of two-dimensional structures parallel to (010), at *y*~0.25, 0.75 (Fig 2 b). A weak S···S contact (T1:E3) helps to connect the columns, though the strongest link is in fact mediated by the second substructure of A, through interactions of the *B···A···B* type to be discussed below.

As opposed to *B*, molecule *A* displays a complex survey of rather long (and correspondingly, weak) contacts, which are relevant as effective interactions could have been regarded with suspicion under normal circumstances. Inspection of Fig. 3a, however, contradicts this view: a striking directionality displayed, assisting the construction of a planar array parallel to (010), is apparent and suggests a cooperative effect of the otherwise very weak interactions involved. Fig 3a shows them in detail: two S···S (T1:E1,E2) (*a*); a C—H···π (T2:E1) (*b*) and a S···π (T3:E2) (*c*), contacts. All of them are extremely weak and, as stated, under normal circumstances, they would have been disregarded. Particularly interesting are those referred to as (*a*) above: they are borderline when commonly accepted standards were applied, *viz*., in the CSD the standard Van der Waal's radius for sulfur is given as 1.80 Å, which would create an upper threshold of 3.60 Å to this type of S···S interactions, with a statistical maximum centred at d=3.586 Å. Inspection of Fig 3a suggests, however, directionality in many S···S contacts with d > 3.60 Å, indicating a possible effect on the two-dimensional organization of the molecules in the solid state. The same can be assessed for the remaining two interactions (*b*) and (*c*). The conclusion is certainly not novel, (see, for instance, Desiraju and Steiner, 1999) and could be summarized in that very weak interactions in the presence of stronger, dominant ones can be safely disregarded but if in isolation and acting in a collective fashion they might strengthen each other, enhancing their overall effects to the extent to sustain well defined substructures, as in the present case.

Coming back to the structural description, *A* planes evolve parallel to, and midway from *B* ones, along (010) at *y*~ 0.0, 0.50 (Fig 3 b). Both substructures are interconnected (Fig 4) by two shorter (stronger) and two longer (weaker) S···S contacts (T1:E4,E5 and T1:E6,E7, respectively). The final result is an extremely even spatial distribution of these cooperative interactions (Figs 2, 3 and 4) providing to the organization of a solid and stable three-dimensional structure.

Trying to assess the general significance of these weak S···S interactions we searched the CSD (Allen, 2002) looking for small molecules, closely related to the title compound. We ended up with two structures built around a 1,3,4-thiadiazole-2(3*H*)-thione ring, *viz*, 2,5-dimercapto-thiadiazole (Bats, 1976; (II)) and 2-thioxo-5-(ethylthio)-3*H*-1,3,4-thiadiazole. (Mrozek *et al.*, 2000; (III)) which differ from the title compound just in the substitutents on the ring (Fig. 5). Even if the packing geometries are different, mainly affected by the diversity of the remaining donors and acceptors present, all three structures basically show a similar survey of S···S contacts with a clear directionality but longer than the usually accepted threshold. Table 4 shows the shortest S···S contacts in all three structures. We conclude that these similarities are in fact a trend, confirming the significance of the analyzed interactions.

### Experimental

As mentioned in the comment section, the formation of the title compound in crystalline form was a serendipitous process, the result of an unsuccesfull attempt to prepare a Mo(VI) complex with the Schiff base ligand *N*'-[bis-(4-amino-phenyl)-methylene]-*N*-methyl-hydrazinecarbodithioic acid methyl ester, (*L*) prepared by condensation of 4,4-diaminobenzophenone and *N*-methyl-*S*-methyldithiocarbazate. After a number of steps, a greenish product, initially presumed to be the *L*-Mo(VI) complex, but later confirmed to be the title compound, was obtained. Since it is hard to relate the small molecule obtained with any of the starting materials or with possible degradation products, the mechanism through which it could have been formed remains unclear, and for this reason we are including herein a detailed description of the steps taken during the synthesis process:

Step-1. Synthesis of *L*: A hot solution of 4,4'-diaminobenzophenone (10 mmol) in 40 ml absolute ethanol was mixed with a similar one of *N*-methyl-*S*-methyldithiocarbazate (10 mmol) in 40 ml of the same solvent. The mixture was refluxed for 6 hs on a water bath. After reducing the volume, an off white product appeared which was filtered off. This product was washed with ethanol several times (5 ml each wash) and dried in a vacuum desiccator over silica gel. Yield: 1.69 g;

Step-2. Attempted preparation of the dioxomolybdeum(VI) complex: Molybdenyl acetylacetonate [MoO_2_(acac)_2_] (3.27 g, 10 mmol) was dissolved in 40 ml dry ethanol, to which a hot solution of the Schiff base ligand, *L*, (3.26 g, 10 mmol) in 40 ml dry ethanol was added. The mixture was refluxed for 6 hs on a water bath. After reducing the volume and keeping standing overnight, a light greenish product appeared which was washed with ethanol several times and dried in a vacuum desiccator over silica gel.

Step-3. Crystallization: The product obtained in Step-2 was allowed to crystallize by slow evaporation from an ethanol-petroleum ether mixture (2:1 *v*/*v*, 10 ml ethanol: 5 ml petroleum ether) solution, to give green crystals, later identified as the title compound.

### Refinement

Methyl groups were idealized (C—H = 0.96 Å) and hydrogen atoms were allowed to ride on their carbon carrier. In all cases, H-atom displacement parameters were taken as *U*_iso_(H) = 1.5*U*_eq_(C).

### Crystal data

**Table:**

C_4_H_6_N_2_S_3_	*F*(000) = 736
*M**_r_* = 178.30	*D*_x_ = 1.539 Mg m^−^^3^
Monoclinic, *P*2_1_/*c*	Mo *K*α radiation, λ = 0.71073 Å
Hall symbol: -P 2ybc	Cell parameters from 6608 reflections
*a* = 9.3505 (4) Å	θ = 3.5–29.0°
*b* = 22.4118 (9) Å	µ = 0.88 mm^−^^1^
*c* = 7.6682 (5) Å	*T* = 295 K
β = 106.661 (5)°	Prism, colourless
*V* = 1539.50 (14) Å^3^	0.3 × 0.2 × 0.2 mm
*Z* = 8	

### Data collection

**Table:**

Oxford Diffraction Gemini CCD S Ultra diffractometer	3821 independent reflections
Radiation source: fine-focus sealed tube	2690 reflections with *I* > 2σ(*I*)
Graphite monochromator	*R*_int_ = 0.060
ω scans, thick slices	θ_max_ = 29.1°, θ_min_ = 3.5°
Absorption correction: multi-scan (*CrysAlis PRO*; Oxford Diffraction, 2009)	*h* = −12→12
*T*_min_ = 0.81, *T*_max_ = 0.84	*k* = −30→29
32965 measured reflections	*l* = −10→10

### Refinement

**Table:**

Refinement on *F*^2^	Primary atom site location: structure-invariant direct methods
Least-squares matrix: full	Secondary atom site location: difference Fourier map
*R*[*F*^2^ > 2σ(*F*^2^)] = 0.040	Hydrogen site location: inferred from neighbouring sites
*wR*(*F*^2^) = 0.102	H-atom parameters constrained
*S* = 1.04	*w* = 1/[σ^2^(*F*_o_^2^) + (0.0398*P*)^2^ + 0.5703*P*] where *P* = (*F*_o_^2^ + 2*F*_c_^2^)/3
3821 reflections	(Δ/σ)_max_ = 0.001
167 parameters	Δρ_max_ = 0.25 e Å^−^^3^
0 restraints	Δρ_min_ = −0.27 e Å^−^^3^

### Special details

**Table:**

Geometry. All e.s.d.'s (except the e.s.d. in the dihedral angle between two l.s. planes) are estimated using the full covariance matrix. The cell e.s.d.'s are taken into account individually in the estimation of e.s.d.'s in distances, angles and torsion angles; correlations between e.s.d.'s in cell parameters are only used when they are defined by crystal symmetry. An approximate (isotropic) treatment of cell e.s.d.'s is used for estimating e.s.d.'s involving l.s. planes.
Refinement. Refinement of *F*^2^ against ALL reflections. The weighted *R*-factor *wR* and goodness of fit *S* are based on *F*^2^, conventional *R*-factors *R* are based on *F*, with *F* set to zero for negative *F*^2^. The threshold expression of *F*^2^ > σ(*F*^2^) is used only for calculating *R*-factors(gt) *etc*. and is not relevant to the choice of reflections for refinement. *R*-factors based on *F*^2^ are statistically about twice as large as those based on *F*, and *R*- factors based on ALL data will be even larger.

### Fractional atomic coordinates and isotropic or equivalent isotropic displacement parameters (Å^2^)

**Table:**

	*x*	*y*	*z*	*U*_iso_*/*U*_eq_	
C3	0.3939 (3)	0.57279 (10)	0.1948 (4)	0.0553 (6)	
H3A	0.4718	0.5836	0.3019	0.083*	
H3B	0.3252	0.6054	0.159	0.083*	
H3C	0.4366	0.5637	0.098	0.083*	
C4	−0.1404 (3)	0.54132 (12)	0.2162 (4)	0.0595 (7)	
H4A	−0.0915	0.5686	0.3112	0.089*	
H4B	−0.2458	0.5414	0.2022	0.089*	
H4C	−0.1228	0.5534	0.1041	0.089*	
C7	0.0292 (3)	0.67498 (11)	0.0189 (4)	0.0588 (7)	
H7A	−0.0033	0.6733	0.1267	0.088*	
H7B	0.0849	0.6396	0.011	0.088*	
H7C	−0.0561	0.6776	−0.0863	0.088*	
C8	0.5833 (3)	0.70055 (13)	0.0599 (4)	0.0654 (7)	
H8A	0.57	0.6818	0.1668	0.098*	
H8B	0.6867	0.6988	0.0635	0.098*	
H8C	0.5243	0.6801	−0.0464	0.098*	
S3	0.55055 (7)	0.44639 (3)	0.29305 (10)	0.05657 (19)	
S1	0.24366 (7)	0.42026 (3)	0.32098 (9)	0.04892 (17)	
S2	−0.06774 (7)	0.46747 (3)	0.27437 (9)	0.05080 (17)	
N1	0.3150 (2)	0.52053 (8)	0.2336 (3)	0.0406 (4)	
N2	0.1669 (2)	0.52810 (8)	0.2290 (3)	0.0420 (4)	
C1	0.3770 (2)	0.46736 (9)	0.2779 (3)	0.0404 (5)	
C2	0.1153 (2)	0.47832 (10)	0.2715 (3)	0.0414 (5)	
S6	−0.09695 (7)	0.80636 (3)	0.00218 (11)	0.0622 (2)	
S4	0.22529 (7)	0.83027 (3)	0.03346 (10)	0.05732 (19)	
S5	0.52524 (7)	0.77719 (3)	0.05225 (10)	0.0598 (2)	
N3	0.1238 (2)	0.72729 (8)	0.0269 (3)	0.0432 (4)	
N4	0.2722 (2)	0.71785 (8)	0.0383 (3)	0.0469 (5)	
C5	0.0747 (2)	0.78324 (10)	0.0200 (3)	0.0427 (5)	
C6	0.3387 (3)	0.76844 (10)	0.0417 (3)	0.0445 (5)	

### Atomic displacement parameters (Å^2^)

**Table:**

	*U*^11^	*U*^22^	*U*^33^	*U*^12^	*U*^13^	*U*^23^
C3	0.0555 (15)	0.0384 (13)	0.0799 (18)	−0.0036 (11)	0.0321 (14)	0.0124 (12)
C4	0.0447 (14)	0.0685 (17)	0.0683 (17)	0.0017 (12)	0.0212 (13)	0.0040 (13)
C7	0.0499 (14)	0.0406 (13)	0.0840 (19)	−0.0117 (11)	0.0164 (13)	−0.0036 (12)
C8	0.0453 (14)	0.0712 (18)	0.083 (2)	0.0059 (13)	0.0242 (14)	0.0050 (15)
S3	0.0457 (3)	0.0504 (4)	0.0778 (5)	0.0068 (3)	0.0244 (3)	0.0075 (3)
S1	0.0477 (3)	0.0377 (3)	0.0634 (4)	−0.0060 (2)	0.0192 (3)	0.0043 (3)
S2	0.0418 (3)	0.0538 (4)	0.0603 (4)	−0.0103 (3)	0.0203 (3)	−0.0044 (3)
N1	0.0373 (9)	0.0368 (10)	0.0511 (11)	−0.0027 (8)	0.0179 (8)	0.0035 (8)
N2	0.0384 (10)	0.0408 (10)	0.0489 (11)	−0.0009 (8)	0.0158 (8)	0.0011 (8)
C1	0.0428 (12)	0.0364 (11)	0.0439 (12)	−0.0031 (9)	0.0155 (10)	0.0012 (9)
C2	0.0426 (12)	0.0432 (12)	0.0402 (12)	−0.0044 (10)	0.0146 (10)	−0.0048 (9)
S6	0.0477 (4)	0.0524 (4)	0.0906 (5)	0.0060 (3)	0.0264 (4)	−0.0029 (3)
S4	0.0507 (4)	0.0375 (3)	0.0862 (5)	−0.0065 (3)	0.0235 (3)	−0.0008 (3)
S5	0.0433 (3)	0.0608 (4)	0.0790 (5)	−0.0070 (3)	0.0235 (3)	0.0066 (3)
N3	0.0355 (9)	0.0385 (10)	0.0559 (12)	−0.0022 (8)	0.0134 (9)	−0.0012 (8)
N4	0.0389 (10)	0.0429 (11)	0.0586 (12)	−0.0011 (8)	0.0136 (9)	0.0008 (9)
C5	0.0422 (12)	0.0389 (12)	0.0480 (13)	−0.0049 (10)	0.0148 (10)	−0.0036 (10)
C6	0.0399 (12)	0.0467 (13)	0.0472 (13)	−0.0025 (10)	0.0129 (10)	0.0017 (10)

### Geometric parameters (Å, º)

**Table:**

C3—N1	1.460 (3)	C8—H8C	0.96
C3—H3A	0.96	S3—C1	1.662 (2)
C3—H3B	0.96	S1—C1	1.736 (2)
C3—H3C	0.96	S1—C2	1.737 (2)
C4—S2	1.796 (3)	S2—C2	1.735 (2)
C4—H4A	0.96	N1—C1	1.326 (3)
C4—H4B	0.96	N1—N2	1.385 (2)
C4—H4C	0.96	N2—C2	1.294 (3)
C7—N3	1.459 (3)	S6—C5	1.655 (2)
C7—H7A	0.96	S4—C6	1.735 (2)
C7—H7B	0.96	S4—C5	1.738 (2)
C7—H7C	0.96	S5—C6	1.734 (2)
C8—S5	1.797 (3)	N3—C5	1.331 (3)
C8—H8A	0.96	N3—N4	1.382 (3)
C8—H8B	0.96	N4—C6	1.289 (3)
			
S3···S2^i^	3.6438 (10)	S1···S6^v^	3.3621 (10)
S2···S2^ii^	3.6319 (9)	S1···S5^iv^	3.8332 (11)
S6···S5^iii^	3.7189 (11)	S2···S4^v^	3.8778 (10)
S3···S4^iv^	3.3671 (10)		
			
N1—C3—H3A	109.5	C1—S1—C2	89.52 (10)
N1—C3—H3B	109.5	C2—S2—C4	99.90 (11)
H3A—C3—H3B	109.5	C1—N1—N2	118.46 (17)
N1—C3—H3C	109.5	C1—N1—C3	124.32 (19)
H3A—C3—H3C	109.5	N2—N1—C3	117.18 (17)
H3B—C3—H3C	109.5	C2—N2—N1	109.28 (18)
S2—C4—H4A	109.5	N1—C1—S3	128.21 (17)
S2—C4—H4B	109.5	N1—C1—S1	108.08 (16)
H4A—C4—H4B	109.5	S3—C1—S1	123.71 (13)
S2—C4—H4C	109.5	N2—C2—S2	124.35 (18)
H4A—C4—H4C	109.5	N2—C2—S1	114.66 (17)
H4B—C4—H4C	109.5	S2—C2—S1	120.96 (13)
N3—C7—H7A	109.5	C6—S4—C5	89.64 (11)
N3—C7—H7B	109.5	C6—S5—C8	100.60 (12)
H7A—C7—H7B	109.5	C5—N3—N4	118.36 (18)
N3—C7—H7C	109.5	C5—N3—C7	123.90 (19)
H7A—C7—H7C	109.5	N4—N3—C7	117.72 (18)
H7B—C7—H7C	109.5	C6—N4—N3	109.63 (18)
S5—C8—H8A	109.5	N3—C5—S6	127.83 (17)
S5—C8—H8B	109.5	N3—C5—S4	107.77 (16)
H8A—C8—H8B	109.5	S6—C5—S4	124.40 (14)
S5—C8—H8C	109.5	N4—C6—S5	124.94 (18)
H8A—C8—H8C	109.5	N4—C6—S4	114.57 (17)
H8B—C8—H8C	109.5	S5—C6—S4	120.49 (13)

Symmetry codes: (i) *x*+1, *y*, *z*; (ii) −*x*, −*y*+1, −*z*+1; (iii) *x*−1, *y*, *z*; (iv) −*x*+1, *y*−1/2, −*z*+1/2; (v) −*x*, *y*−1/2, −*z*+1/2.

### Hydrogen-bond geometry (Å, º)

Cg1 is the centroid of the C1,C2,N1,N2,S1 ring.

**Table:**

*D*—H···*A*	*D*—H	H···*A*	*D*···*A*	*D*—H···*A*
C4—H4*C*···*Cg*1^vi^	0.96	2.86	3.589 (3)	134

Symmetry code: (vi) −*x*, −*y*+1, −*z*.

### X-π contacts (X: Cg, S).

**Table:**

G1···G2	d(G1-G2)(Å)	d(G1-G1*)(Å)	<G1*-G1-G2>(°)
Cg2···Cg2^vii^	3.8958 (13)	3.7320 (9)	16.27 (2)
S3···Cg1^viii^	3.7271 (11)	3.5532 (13)	17.66 (2)

Symmetry codes: (vii) x,3/2-y,1/2+z; (viii) 1-x, 1-y, 1-z. Cg1 and Cg2 are the centroids of the C1,C2,N1,N2,S1 and C5,C6,N3,N4,S4 rings, respectively.d(G1-G2): G1-G2 vector length; G1*: projection of the G1 centre onto the G2 plane. <G1*-G1-G2>: angle subtended by the G1*-G1 and G2-G1 vectors.

### Table 4: Comparative table of S···S distances (Å) found in closely related structures I (this work), II (Bats, 1976) and III (Mrozek *et al.*, 2000).

**Table:**

(I)	(II)*	(III)
3.3621 (10)[-0.24]	3.565[-0.04]	3.577 (4)[-0.03]
3.3671 (10)[-0.23]	3.694[+0.09]	3.890 (4)[+0.29]
3.6319( 9)[+0.03]	3.771[+0.17]	3.906 (4)[+0.30]
3.6438 (10)[+0.04]	3.924[+0.32]	3.975 (4)[+0.37]
3.7189 (11)[+0.11]		
3.8332 (11)[+0.23]		
3.8778 (10)[+0.28]		

*: su's not provided in the original work. In square brackets, deviations from the sum of commonly accepted Van der Waals radii (Bondi, 1964).
